# Supplementary material for: Characterizing programmed cell death features in osteoarthritis through integrative multiomics and machine learning analysis
Source: PeerJ. 2025 Oct 10;13:e20104. doi: 10.7717/peerj.20104 (PMC12517281; doi:10.7717/peerj.20104)
Supplement: Supplemental Information 1 [file peerj-13-20104-s001.docx]

| **Recent research on mechanisms of osteoarthritis (OA)** | | | | | |
| --- | --- | --- | --- | --- | --- |
| **Literature** | **Study Objectives** | **Methodology** | **Key Findings** | **Strengths** | **Limitations** |
| Identification and validation of aging related genes in osteoarthritis(Du et al. 2025) | To identify OA associated genes and elucidate molecular pathways, focusing on CX3CR1 as a potential diagnostic biomarker and therapeutic target. | Integrated bioinformatics analysis (GEO datasets, WGCNA, PPI networks) with qPCR, Western blot (WB) and immunohistochemistry (IHC) to explore CX3CR1's role in OA pathogenesis. | CX3CR1 was significantly upregulated in OA, correlating with lipid metabolism, extracellular matrix degradation, and immune cell infiltration (e.g., activated mast cells), and showed diagnostic potential (AUC = 0.806). | Combined multi-omics approaches with robust experimental validation, highlighting CX3CR1's mechanistic link to OA progression and immune modulation. | Reliance on public datasets; lack of detailed molecular mechanism exploration and in vivo therapeutic validation. |
| Identification of WDR74 and TNFRSF12A as biomarkers for early osteoarthritis using machine learning and immunohistochemistry(Chen et al. 2025) | To identify ubiquitination-related genes as diagnostic biomarkers for early OA using machine learning and validate their clinical relevance. | Integrated scRNA-seq, bulk RNA-seq, machine learning (XGBoost), and IHC analyze OA datasets and validate candidate genes (WDR74, TNFRSF12A). | WDR74 and TNFRSF12A were identified as core OA biomarkers, with diagnostic model AUC >0.9, and validated via qRT-PCR/IHC in human OA cartilage and IL-1β-stimulated chondrocytes. | Multi-modal approach combining scRNA-seq, ML, and experimental validation; high diagnostic accuracy and subgroup stratification potential. | Requires larger cohort validation; functional roles of WDR74/TNFRSF12A in OA pathogenesis need further mechanistic studies. |
| Identification of Energy Metabolism-Related Subtypes and Diagnostic Biomarkers for Osteoarthritis(Xu et al. 2025) | To identify energy metabolism-related genes as diagnostic biomarkers for OA and explore their association with immune infiltration | Integrated bioinformatics (GEO datasets, differential expression analysis, PPI networks) with machine learning (LASSO, SVM-RFE, RF) and immune infiltration analysis (CIBERSORT, ssGSEA). | Identified NUP98 and RPIA as diagnostic biomarkers (AUC >0.9), validated in training (GSE51588) and test sets (GSE63359), with distinct immune infiltration patterns in OA subtypes. | Multi-modal approach combining transcriptomics, machine learning, and immune profiling; robust diagnostic performance validated across datasets. | Limited clinical validation; mechanistic roles of NUP98/RPIA in OA pathogenesis require further experimental investigation. |
| Identification of Genes Linked to Meniscal Degeneration in Osteoarthritis: An In Silico Analysis(Papageorgiou et al. 2025) | To identify key genes and pathways involved in meniscal degeneration during (OA progression through computational analysis of transcriptomic data. | Integrated bioinformatics (DESeq2, PPI networks, GO/KEGG enrichment) with comparative analysis of OA meniscus, synovium, and cartilage datasets (GSE185064, GSE114007, GSE143514). | Identified 85 mRNAs (e.g., MMP13, RUNX2, ITGB2) and 8 lncRNAs (e.g., XIST, H19) dysregulated in OA meniscus;GJB, PAQR5, and CLEC12A were shared across all OA-affected tissues. | Multi-tissue comparative approach revealing shared pathogenic mechanisms; robust functional enrichment linking genes to inflammation, ECM degradation, and lipid metabolism. | Small cohort size; lack of experimental validation; age/gender metadata gaps in datasets may confound results. |
| Identification of Key Chondrocyte Apoptosis-Related Genes in Osteoarthritis(Wang et al. 2025) | To identify key chondrocyte apoptosis-related genes (CARGs) in OA through bioinformatics and experimental validation, focusing on their diagnostic and therapeutic potential. | Integrated WGCNA, PPI network analysis, and in vitro assays (IL-1β-stimulated C28/I2 chondrocytes) to validate hub genes (NFKB1, ICAM1) from GEO datasets (GSE32317, GSE55235). | Identified NFKB1 and ICAM1 as hub CARGs in OA, upregulated in IL-1β-induced chondrocytes and correlated with apoptosis, ECM degradation, and inflammation (AUC: 0.9 for ICAM1). | Multi-modal approach combining bioinformatics (WGCNA, ROC analysis) with experimental validation (qRT-PCR, WB) to confirm gene roles in OA pathogenesis. | Lack of in vivo validation; clinical relevance of NFKB1/ICAM1 as therapeutic targets requires further preclinical studies. |
| Identification of ZNF652 as a Diagnostic and Therapeutic Target in Osteoarthritis Using Machine Learning(Chen et al. 2024) | To identify key chondrocyte apoptosis-related genes (CARGs) in OA through bioinformatics and experimental validation, focusing on their diagnostic and therapeutic potential. | Integrated WGCNA, PPI network analysis, and in vitro assays (IL-1β-stimulated C28/I2 chondrocytes) to validate hub genes (NFKB1, ICAM1) from GEO datasets (GSE32317, GSE55235). | Identified NFKB1 and ICAM1 as hub CARGs in OA, upregulated in IL-1β-induced chondrocytes and correlated with apoptosis, ECM degradation, and inflammation (AUC: 0.9 for ICAM1). | Multi-modal approach combining bioinformatics (WGCNA, ROC analysis) with experimental validation (qRT-PCR, Western blot) to confirm gene roles in OA pathogenesis. | Lack of in vivo validation; clinical relevance of NFKB1/ICAM1 as therapeutic targets requires further preclinical studies. |
| Identification and Validation of Aging-Related Genes in Osteoarthritis(Du et al. 2025) | To identify aging-related genes (ARGs) linked to osteoarthritis (OA) pathogenesis using bioinformatics and experimental validation, focusing on synovial tissue to uncover diagnostic and therapeutic targets. | Integrated GEO datasets (GSE55235, GSE55457, GSE82107) for DEG screening, ARG intersection, PPI networks, machine learning (LASSO/RF), and qRT-PCR validation in OA synovial tissues. | Identified ATF3, KLF4, NFKBIA, and SOD2 as downregulated ARGs in OA, correlating with immune infiltration (e.g., altered Mast cells, M1 macrophages) and oxidative stress pathways (AUC: 0.967 for diagnostic model). | Multi-dataset validation, machine learning robustness, and experimental confirmation via qRT-PCR, highlighting translational potential for OA biomarkers. | Small clinical sample size; lack of in vivo mechanistic studies and single-cell resolution for synovial cell-type-specific gene expression. |
| Identification of Key Genes in Osteoarthritis Development: Biomarker Discovery and Therapeutic Targets(Wu et al. 2025) | To identify synovial tissue-derived diagnostic biomarkers (CXCL8, CXCL2, DUSP5, TNFSF11) for OA through multi-dataset bioinformatics analysis and experimental validation, addressing the limitations of single-biomarker approaches. | Integrated GEO datasets (GSE1919/GSE55235/GSE82107) for DEG screening, PPI network construction (Cytoscape), machine learning (ROC AUC >0.8), and qRT-PCR validation in 5 OA/control synovial samples. | Identified 4 hub genes: CXCL8 (AUC=1.0), CXCL2 (AUC=0.901), DUSP5 (AUC=0.851), and TNFSF11 (AUC=0.810), linking synovial inflammation (CXCL8/2) and bone remodeling (TNFSF11/DUSP5) pathways. | Multi-dataset validation (training + GSE29746), synergistic biomarker clusters overcoming single-target limitations, and drug prediction (e.g., acetaminophen-DUSP5 interaction). | Small clinical cohort (n=5/group), lack of mechanistic studies, and platform heterogeneity across GEO datasets. |
| Identification of MEG3 and MAPK3 as Potential Therapeutic Targets for Osteoarthritis Through Multiomics Integration and Machine Learning(Ma et al. 2025) | To identify causal genes for knee osteoarthritis (KOA) using Mendelian randomization (MR), transcriptomics, and machine learning, focusing on synovial tissue biomarkers with diagnostic and therapeutic potential | Identified MEG3 (protective, OR=0.962) and MAPK3 (risk, OR=1.094) as diagnostic biomarkers, linked to immune infiltration (e.g., Tfh/mast cells) and validated in synovial tissues (AUC >0.7 in training/validation sets). | Identified MEG3 (protective, OR=0.962) and MAPK3 (risk, OR=1.094) as diagnostic biomarkers, linked to immune infiltration (e.g., Tfh/mast cells) and validated in synovial tissues (AUC >0.7 in training/validation sets). | Multi-modal integration (MR + transcriptomics + machine learning), robust cross-validation, and non-invasive biomarker potential via blood-based eQTL associations. | Small clinical validation cohort (n=3/group), lack of mechanistic studies, and unaddressed subclinical OA in controls. |
| Integrating Bioinformatics and Machine Learning to Identify Biomarkers of Branched Chain Amino Acid Related Genes in Osteoarthritis(ZhaYang et al. 2025) | To identify BCAA-related biomarkers (SLC3A2 and SLC7A5) in OA through multi-omics integration, machine learning, and experimental validation, addressing gaps in early diagnosis and metabolic dysregulation. | Combined GEO transcriptomics (GSE114007/GSE51588), differential expression analysis, WGCNA, three machine learning algorithms (LASSO/SVM-RFE/Boruta), and experimental validation (qPCR/IHC/WB) to screen and validate biomarkers. | Identified SLC3A2 and SLC7A5 as diagnostic biomarkers (AUC >0.7), linked to ribosome/insulin pathways and regulated by XIST/OIP5-AS1 miRNAs; nomogram model achieved 98.1% accuracy in OA prediction. | Multi-algorithm cross-validation, robust nomogram predictive performance, and experimental validation (qPCR/IHC) supporting clinical relevance of BCAA metabolism in OA. | Small clinical cohort (n=10/group), reliance on cartilage-specific datasets, and unvalidated mechanistic roles of SLC3A2/SLC7A5 in BCAA transport. |
| Identification of Ferroptosis-Related Genes and Potential Drugs in Osteoarthritis(Song et al. 2025) | To identify ferroptosis-related biomarkers (GPX4, TFRC, SLC7A11, EGFR, IL1B) in OA and elucidate resveratrol’s therapeutic mechanism via ferroptosis modulation. | Integrated GEO transcriptomics (GSE55235/GSE77298/GSE82107), WGCNA, PPI networks, immune infiltration analysis, molecular docking, and in vitro validation (qPCR/WB) to screen targets and validate resveratrol’s effects. | Identified 462 ferroptosis-related genes in OA; resveratrol ameliorated OA by targeting GPX4/SLC7A11 to suppress ferroptosis and inflammation via PI3K-Akt/mTOR pathways (p<0.001). | Multi-omics validation (bioinformatics + in vitro experiments), robust immune infiltration insights, and molecular docking confirming resveratrol-target interactions. | Small clinical cohort (n=10/group), lack of in vivo validation, and unaddressed subclinical OA in controls. |
| Screening of Potential Biomarkers of Osteoarthritis: A Bioinformatics Analysis(Hou et al. 2024) | To identify novel diagnostic biomarkers (JUN, ATF3, DUSP1) and regulatory mechanisms (hsa-mir-26b-5p) in OA through integrative bioinformatics analysis. | Analyzed GEO datasets (GSE55457/GSE55235/GSE114007) for DEGs, performed GO/KEGG enrichment, constructed PPI networks, validated hub genes via ROC curves, and predicted miRNA interactions (hsa-mir-26b-5p). | Identified JUN, ATF3, and DUSP1 as downregulated hub genes (AUC >0.95) linked to MAPK signaling and mitochondrial dysfunction; hsa-mir-26b-5p potentially regulates all three genes. | Multi-dataset validation, robust ROC performance (AUC up to 1.00), and integration of miRNA-mRNA networks revealing hsa-mir-26b-5p as a therapeutic target. | Lack of in vitro/in vivo validation and clinical cohort data to confirm biomarker specificity in diverse OA subtypes. |

**Reference：**

Chen Y, Liang R, Zheng X, Fang D, Lu WW, and Chen Y. 2024. Identification of ZNF652 as a Diagnostic and Therapeutic Target in Osteoarthritis Using Machine Learning. *J Inflamm Res* 17:10141-10161. 10.2147/jir.s488841

Chen Y, Lin J, Shi D, Miao Y, Xue F, Liu K, Wang X, and Zhang C. 2025. Identification of WDR74 and TNFRSF12A as biomarkers for early osteoarthritis using machine learning and immunohistochemistry. *Front Immunol* 16:1517646. 10.3389/fimmu.2025.1517646

Du J, Zhou T, Dong Y, Sun Y, and Peng W. 2025. Identification and validation of aging related genes in osteoarthritis. *Front Genet* 16:1561644. 10.3389/fgene.2025.1561644

Hou Y, Yang Z, Ma J, Liu J, Sun X, Li J, and Zhang B. 2024. Screening of potential biomarkers of osteoarthritis: a bioinformatics analysis. *Clin Rheumatol*. 10.1007/s10067-024-07213-x

Ma B, Wang X, Xu C, Xu Z, Zhang F, and Cheng W. 2025. Identification of MEG3 and MAPK3 as potential therapeutic targets for osteoarthritis through multiomics integration and machine learning. *Sci Rep* 15:23240. 10.1038/s41598-025-06175-7

Papageorgiou AA, Balis C, and Papathanasiou I. 2025. Identification of Genes Linked to Meniscal Degeneration in Osteoarthritis: An In Silico Analysis. *Int J Mol Sci* 26. 10.3390/ijms26146651

Song C, Shen B, Chen C, Yang L, Zhang C, Liu F, Chen F, and Wu X. 2025. Identification of ferroptosis-related genes and potential drugs in osteoarthritis. *Inflamm Res* 74:70. 10.1007/s00011-025-02040-5

Wang W, Hong J, Cao T, Ye F, Gao J, and Qin S. 2025. Identification of Key Chondrocyte Apoptosis-Related Genes in Osteoarthritis Based on Weighted Gene Co-Expression Network Analysis and Experimental Verification. *Crit Rev Immunol* 45:15-29. 10.1615/CritRevImmunol.2024051935

Wu L, Wen D, Wang W, Wang Y, and Zhang L. 2025. Identification of key genes in osteoarthritis development: biomarker discovery and therapeutic targets. *Front Med (Lausanne)* 12:1518580. 10.3389/fmed.2025.1518580

Xu S, Ye J, and Cai X. 2025. Identification of Energy Metabolism-Related Subtypes and Diagnostic Biomarkers for Osteoarthritis by Integrating Bioinformatics and Machine Learning. *J Multidiscip Healthc* 18:1353-1369. 10.2147/jmdh.s510308

ZhaYang XZ, Chen YX, Hua WD, Bai ZL, Jin YP, Zhao XW, Liu QF, and Meng ZD. 2025. Integrating bioinformatics and machine learning to identify biomarkers of branched chain amino acid related genes in osteoarthritis. *BMC Musculoskelet Disord* 26:517. 10.1186/s12891-025-08779-6
